# Sex-Specific Regulatory Systems for Dopamine Production in the Honey Bee

**DOI:** 10.3390/insects13020128

**Published:** 2022-01-25

**Authors:** Ken Sasaki, Tomohiro Watanabe

**Affiliations:** Graduate School of Agriculture, Tamagawa University, Machida, Tokyo 194-8610, Japan; wtnbt4ba@agrs.tamagawa.ac.jp

**Keywords:** *Apis mellifera*, biogenic amine, brain, dopamine, male, reproduction, sex, social insect

## Abstract

**Simple Summary:**

In this review, we describe sex-specific differences in the regulatory systems for dopamine production in the brains of social insects, focusing on the honey bee. Dopamine has a crucial role in the promotion of reproduction in both sexes of the honey bee and is a key substance for understanding the mechanisms underlying the reproductive division of labor in females. Studies associated with dopamine regulation have been performed mainly in females, with less of a focus on its regulation in males. In social insects, males are specialized for reproduction and do not exhibit division of labor; however, they have evolved to adapt their social system and have acquired/discarded physiological and behavioral characteristics. Therefore, studies exploring the dopaminergic system in males can contribute to our understanding of social adaptation in males. We integrate findings related to dopamine in both honey bee sexes and provide insights into the physiology involved in dopaminergic systems in social insects.

**Abstract:**

Dopamine has multiple functions in the modulation of social behavior and promotion of reproduction in eusocial Hymenoptera. In the honey bee, there are sex-specific differences in the regulation of dopamine production in the brain. These different dopaminergic systems might contribute to the maintenance of sex-specific behaviors and physiology. However, it is still not fully understood how the dopaminergic system in the brain is regulated by endocrinal factors and social stimuli in the colony. In this review, we focus on the regulation of dopamine production in queens, workers, and males in the honey bee. Dopamine production can be controlled by queen substance, juvenile hormone, and exogenous tyrosine from food. Queens can control dopamine production in workers via queen substance, whereas workers can manipulate the supply of tyrosine, a precursor of dopamine, to queens and males. The regulation of dopamine production through social interaction might affect the reproductive states of colony members and maintain sex-specific behaviors in unpredictable environments.

## 1. Introduction

The division of labor in eusocial insects is fundamental to the organization of insect societies. In highly eusocial hymenopterans, including the honey bee, females are differentiated both behaviorally and morphologically into reproductive individuals (queens) and infertile helper individuals (workers). These polymorphic individuals are called ‘castes’, and caste differentiation occurs initially based on nutrition during the larval stage [1,2,3]. Such differentiation creates not only caste-specific external morphology, but also caste-specific internal organs and physiology. The brain is also specialized to each caste during metamorphosis and is adapted both morphologically and physiologically to perform each behavioral task required of that caste [4,5,6,7,8]. Workers engage in various tasks, including nursing the brood; cleaning, constructing, and guarding the nest; and foraging. Some species, such as honey bees, change from performing tasks inside the nest to outside the nest with age [2,9], whereas others, such as ants [10] or bumble bees [5,11], perform tasks based on their external morphology. How the caste-specific behaviors of adults are physiologically regulated is an important issue in the division of labor in eusocial insects.

Males in eusocial hymenopteran species do not exhibit division of labor and are cared for by workers. Honey bee males are fed by nurse bees during their early adult stage before sexual maturity [2,12]. Even after sexual maturation, males consume honey, both as food and fuel, depending on the food storage levels in the colony. The role of sexually mature males (i.e., more than 8 days old) is to fly and mate with virgin queens. Males repeatedly fly to drone congregation areas to mate with virgin queens, but return to their nests if they do not mate successfully [12,13]. Thus, males have lost several behavioral characteristics that are generally observed in solitary males, such as foraging behavior. Meanwhile, they have also acquired the behavioral characteristics for returning to the nest in orientation flight learning [14]. They might have adapted to their original social system in terms of their life history and acquired physiological characteristics related to sociality. Thus, exploration of the behavioral physiology of male honey bees contributes to understanding the evolution of their behavior in a highly social environment; however, few papers have focused on behavioral physiology in male honey bees.

Insect hormones, including juvenile hormone (JH), contribute to caste differentiation via nutrition-sensing signal cascades during the larval stage and maintain the physiological state of the central nervous system and peripheral organs of the adults [3,15,16,17]. These hormones are considered to act broadly on receptors in the brain and influence neuroendocrine systems that maintain sex-specific behaviors. Biogenic amines are neuroactive substances controlling behaviors and reproduction in insects [18,19,20,21]. These substances are synthesized in neurosecretory cells in the brain or other ganglia and secreted into the relevant neural circuit and other target tissues. In target cells, a specific amine binds with its receptors, changing the intracellular levels of secondary messengers, including cAMP, and resulting in the expression of relevant genes or changes in threshold for neuronal activation [18,19,20,21]. However, it is still not fully understood how the regulatory systems of these amines control social and sexual behavior in the honey bee.

Dopamine is a biogenic amine that has multiple roles in the regulation of behavior and reproduction in eusocial insects [22,23,24]. It has numerous overlapping functions, acting as a neurotransmitter in locally confined interneuronal signaling, as a neuromodulator in postsynaptic cells in several restricted regions of the central nervous system, or as a neurohormone when released into the hemolymph and transported via the circulatory system to target tissues in the body. Histochemical studies indicate that several groups of dopamine secretion cells exist in the honey bee brain [25,26,27]. These cells project their neuropil to the central body; mushroom body; other areas of the frontal, lateral, and caudal protocerebrum; dorsal deutocerebrum; and antennal lobe [25,26,27]. Dopamine is recognized by specific receptors at the surface of target cells, and receptor binding translates the chemical signal into a specific electrical or biochemical response in the target cell [19,28].

## 2. Dopaminergic System in Females

### 2.1. Caste Differences in the Dopaminergic System in the Brain

Caste differences in the brain levels of dopamine have been reported in adult honey bees [29,30]. The brain levels of dopamine in newly emerged virgin queens are approximately four times higher than those in same-aged workers [30]. There are also caste differences in the levels of dopamine-related substances, suggesting that the dopamine synthesis/metabolic pathway is activated in virgin queens. A mechanism by which caste differences in dopamine levels are maintained is the high expression of genes encoding enzymes involved in dopamine biosynthesis [30] and the additional supply of exogenous dopamine precursors from food [31], resulting in different levels in the brain. Newly emerged queens have higher expression levels of genes encoding tyrosine hydroxylase (*Amth*) and DOPA decarboxylase (*Amddc*) compared with newly emerged workers [30]. Given that the numbers of neurons expressing *Amddc* are almost the same between castes [30], each dopaminergic neuron might enhance the expression levels of *Amth* and *Amddc* in the brain of the queen. However, this possibility has not been confirmed at the individual neuron level. Queens are also fed ‘royal jelly’ by nurse bees; this contains the dopamine precursor, tyrosine [32,33,34]. They are fed royal jelly during the late larval stages and when they are adults, whereas adult workers feed on honey and pollen [2]. It has also been reported that female larvae fed larger amounts of royal jelly become adult females with higher levels of dopamine, tyrosine, and DOPA in the brain [31]. The higher dopamine levels in virgin queens are associated with the activation of queen-specific behaviors, including mating flight, locomotor activity, and fighting with rival virgin queens [24,31].

Caste differences in the dopamine levels in the brain have been reported in the bumble bee *Bombus ignitus* [35]. Newly emerged queens have higher dopamine and dopamine-related substances in the brain compared with same-aged workers. The differences in the dopamine levels are smaller in the bumble bee (approximately two-fold) than in the honey bee (approx. four-fold).

### 2.2. Age-Related Increases in Dopamine in Workers

In workers in queenright colonies, the levels of dopamine in the brain increase with age [36,37,38]. The levels of octopamine and serotonin in the brain also increase with age, suggesting that the production of several functional monoamines is enhanced simultaneously in the brain [36,37]. These increases in the monoamine levels are associated with the transition of tasks as the workers age. JH also increases in the hemolymph with worker age [15,39]. The application of a JH analog enhanced the octopamine levels in the brain [40], although its effects on the brain levels of dopamine or serotonin are unknown. The expression of genes encoding dopamine receptors (*Amdop1*, *Amdop2*, and *Amdop3*) [41] and a dopamine transporter (*Amdat*) [42] increases with age in workers, suggesting that the dopaminergic system, including dopamine production and signaling, is upregulated with age in workers. Factors affecting the dopaminergic system might be age-related substances or neural activities, including experience, but remain to be determined.

### 2.3. Transition of Reproductive States Mediated by Dopamine

Workers are usually infertile with inactivated ovaries in the presence of queens, but have the potential to become reproductive individuals with activated ovaries, laying unfertilized eggs that are destined to become males. This switch is a reproductive tactic, changing from cooperative altruistic behaviors for determining the success of the colony to selfish behaviors for determining their own reproductive success. In the honey bee, dopamine is involved in the reproductive maturation and reproductive behaviors of females [24]. The brain levels of dopamine are correlated with the reproductive status of workers [43]. Dopamine accelerates the ovarian activity of reproductive workers [44]. In addition, positive relationships between the brain dopamine levels and ovarian activity in workers have been reported in bumble bees, paper wasps, and ants [24,45,46]. Ovarian activation in reproductive females by dopamine has also been reported in paper wasps and ants [24]. These observations suggest that brain dopamine is a key substrate in the regulation of reproduction in reproductive individuals of both primitive and highly eusocial Hymenoptera. 

The gene expression of dopamine receptors in the brains of queenless honey bee workers is modulated by the presence of a queen in the colony [41,47]. In the absence of a queen, the expression of *Amdop1* in the brain is enhanced in workers in cages without a brood [41], whereas in workers in queenless colonies with a brood, the expression of *Amdop1* and *Amdop2* is reduced [47]. In the ovaries, the expression of *Amdop1* is enhanced, whereas that of *Amdop3* is reduced in workers in queenless colonies [47]. The function of these receptors in the brain and ovaries should be tested by the application of dopamine receptor drugs or treatment with RNA interference targeting a particular dopamine receptor gene.

## 3. Dopaminergic System in Males

Male honey bees mate with a queen while flying; therefore, mating flight activity is an important factor for their mating success. Flight activity gradually increases with age [48] and males begin their orientation flights when they are 6–8 days old [14]. The reproductive organs of males also mature once they are 8 days old [13,49]. 

Dopamine can contribute to the elevation of mating flight activity in males. Its levels in the brain, thoracic ganglia, and hemolymph increase up to 7–8 days of age [48]. The expression of the dopamine transporter gene *Amdat* increases progressively for at least 15 days after emergence [42]. The expression of *Amdop1*, *Amdop2*, and *Amdop3* is also enhanced up to 8 days of age [50], suggesting the age-related regulation of dopaminergic systems. Locomotor activities also increase with age and are enhanced by a dopamine-receptor agonist (6,7-ADTN) and inhibited by the antagonist flupentixol [48]. Flight-initiation and flight-maintaining activities are also enhanced by dopamine injections [51]. Given that the hemolymph dopamine levels change in parallel with those in the brain, dopamine circulating in the hemolymph acts on peripheral tissues involved in mating flights and copulation. In fact, the male reproductive organs comprising the testis, seminal vesicle, and mucus gland express dopamine receptor genes [49]. In seminal vesicles, four dopamine receptor genes, *Amdop1*, *Amdop2*, *Amdop3*, and *Amgpcr19* (encoding the dopamine-ecdysteroid receptor) [19,28,52], are strongly expressed and the tissue increases the cAMP levels in response to dopamine [49]. However, the functional differences between these receptors in the seminal vesicles have not been determined.

## 4. Factors Affecting Dopamine Production in the Brain

### 4.1. Queen Substance

**Figure 1:**
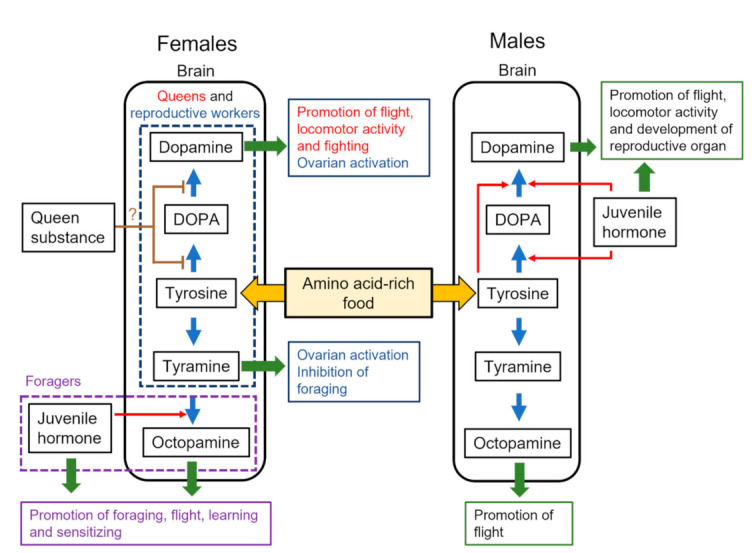
Sex-specific regulation of monoamine production from tyrosine in females and males. Arrows in blue and red indicate the synthetic pathways of biogenic amines and promotion of monoamine production, respectively. Arrows in green indicate the modulation of behavior and physiology. Brown lines indicate potential inhibitory effects on monoamine production. Different-colored letters indicate the behavioral effects of biogenic amines on females with different behavioral states (queens, reproductive workers, and foragers).

In terms of pheromones, the queen substance can control the behavior and physiology of workers in highly eusocial Hymenoptera. In the honey bee, a queen mandibular pheromone (QMP) inhibits ovarian activity in workers [53,54] and homovanillyl alcohol (HVA) reduces the brain dopamine levels [41] (Figure 1). However, how HVA controls the dopamine synthetic pathway remains to be determined. HVA has the potential to bind a dopamine receptor (AmDOP3) and acts as an agonist [55]. It can control brain dopamine in two ways: via the detection of HVA by antennae and the transmission of neural signals to influence the brain dopamine levels, or via the oral intake of HVA, which then acts directly on the dopaminergic system in the brain via the hemolymph. The former is the more plausible mechanism and is supported by neural responses to HVA in the antennal lobes [56]. Signals from HVA detected by the antennae in workers are processed through particular areas of the antennal lobes and sent to the mushroom body mainly via the lateral antennal lobe tract [56]. This pathway is one of the candidates for a QMP-processing pathway to inhibit reproduction and trigger cooperative altruistic behaviors for the success of the colony. However, more evidence for the latter is required. In other species, substances including long-chain hydrocarbons and esters suppress ovarian activity in workers [57]. The long-chain hydrocarbons are detected by the antennae [58,59] and can influence brain dopamine levels in eusocial hymenopterans. In males, the effects of dopamine production by QMP are unknown, but such details are required for a complete picture of sex-specific dopamine production in the honey bee.

### 4.2. Juvenile Hormone

The dopamine levels in the brain, thoracic ganglia, and hemolymph of males change in parallel with those of JH titer in the hemolymph or JH synthesis in the corpora allata [48,60,61]. JH triggers the onset of mating flights in males [61,62,63] and has a role similar to that of dopamine. The application of a JH analog (methoprene) to immature males selectively enhanced the levels of dopamine in the male brain [50,51] (Figure 1) and the expression of genes encoding enzymes (*Amth* and *Amddc*) involved in dopamine biosynthesis [64], but not the levels of octopamine in the male brain [51]. Methoprene also selectively enhances the expression of *Amdop1* in the male brain [50], suggesting that JH influences both dopamine production and signaling. The relationship between JH and dopamine has also been reported in males of the large carpenter bee *Xylocopa appendiculata* [65], suggesting that the relationship is an ancestral characteristic in male Hymenoptera. In other solitary species, the JH–dopamine relationship has been reported in the fruit fly *Drosophila melanogaster* [66,67,68] and plant bug *Lygus Hesperus* [69]. In *D. melanogaster*, the roles of JH in reproduction with dopamine are different between sexes: the activation of the ovaries in females and the modulation of courtship behavior in males [67,68]. Thus, the JH–dopamine relationship and sexual dimorphism of the JH reproductive function are shared among solitary species across different orders.

In workers under queenright conditions, JH enhances the octopamine levels via the expression of *Amtbh* (tyramine β-hydroxylase) in the brain, resulting in the promotion of foraging behavior [40,70,71] (Figure 1), but the effects of JH on the dopamine levels in the brain are unknown. Under queenless conditions, reproductive workers increase the dopamine levels in their brains, but maintain low JH titers in the hemolymph [72,73]. Queens also show low JH titers in the hemolymph [72], suggesting fewer effects of JH on dopamine production.

### 4.3. Tyrosine Intake

Food consumption can be influenced by the presence of a queen and broods in a colony and affect the supply of biogenic amine precursors. In the honey bee, tyrosine, a common precursor of dopamine and tyramine (Figure 1), is contained in royal jelly [32,33,34]. Royal jelly contains at least 26 amino acids, of which tyrosine is not the most abundant. Royal jelly is fed by nurse bees to the queen and larvae in queenright colonies; however, in queenless colonies without broods, it can be shared among the workers. Therefore, reproductive females in queenless colonies might ingest a relatively large amount of tyrosine by consuming royal jelly-like food. This intake of tyrosine or royal jelly enhances the levels of brain dopamine and tyramine in queenless workers (Figure 1) and accelerates their transition from normal workers to reproductive females [74]. 

In males, the intake of tyrosine or royal jelly also enhances the levels of dopamine and *N*-acetyldopamine [75] (Figure 1). Oral application of tyrosine enhanced the expression of the enzyme-encoding gene *Amddc* involved in dopamine synthesis and resulted in increased dopamine levels in the brain [64]. Given that males less than 3 days old do not take food by themselves and instead depend on food exchange with nurse bees [75,76,77,78], their supply of tyrosine is controlled by the nurse bees. These findings led to the ‘brain amine manipulation hypothesis’, whereby the brain levels of dopamine and other biogenic amines synthesized from tyrosine in males can be manipulated by workers. If nurse workers feed tyrosine-rich food to young males, the males have increased levels of dopamine in their brains, whereas if workers deny such food to males, the latter will have decreased dopamine levels, resulting in the delay of behavioral development or even starvation. In fact, workers distinguish between tyrosine-rich food containing pollen and carbohydrate-rich food containing honey when feeding adults [33,79]. In addition, workers can control the frequency of trophallactic interactions and the amount of food given to males depending on the availability of food within the colony [80]. The tyrosine supply by workers is influenced by the pollen stored in the nests, which might, in turn, depend on the season and location of the nest. 

**Figure 2:**
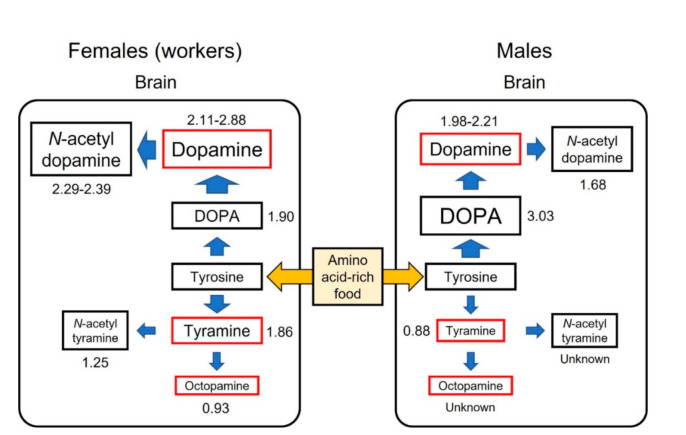
Tyrosine metabolic pathways in the brains of honey bee workers and males. Values beside each amine indicate the ratio of the mean values of amine levels in tyrosine-fed individuals to the mean values of amine levels in control (sucrose-fed) individuals after feeding for 8 days. The ratios indicate the metabolic rates when tyrosine is supplied by diet. Data from [64,74,75] (Table S1).

Dopamine and tyramine are synthesized from tyrosine, although these pathways are independent. The ratio of the mean value of amine levels in tyrosine-fed individuals to the mean value of amine levels in control (sucrose-fed) individuals was calculated from previously published data [64,74,75] (Table S1) and compared between the dopamine and tyramine (or octopamine) pathways (Figure 2). The ratios were larger in the dopamine pathway than in the tyramine pathway in both workers and males, indicating that the metabolic pathway of tyrosine is biased toward dopamine. The synthesis rate of dopamine was faster in workers than in males: workers metabolized tyrosine into larger amounts of dopamine and *N*-acetyldopamine, whereas males converted tyrosine into larger amounts of DOPA (Figure 2). These differences might result from the indirect intake of tyrosine in males due to feeding by nurse bees versus the direct intake of tyrosine by workers.

## 5. Conclusions

Here, we have reviewed the dopaminergic regulation systems in the brains of honey bees. Dopamine has multiple functions in the nervous system that result in behavioral changes and in peripheral tissues that result in physiological changes. Reports of the behavioral effects of dopamine are accumulating in the honey bee and studies addressing dopamine targets in the brain are ongoing. Various peripheral tissues are potential targets of dopamine in reproductive individuals, but only a few studies on the peripheral targets of dopamine have been published [47,49]. Given that the transition to reproductive individuals causes various physiological changes in peripheral tissues, the integration of these tissues for the initiation of reproduction involving hemolymph dopamine will be important. Therefore, future studies should focus on determining the link(s) between the regulatory systems of brain dopamine in response to social environmental factors and dopamine action on sex-specific tissues.

Dopamine production can be manipulated in several ways through social interactions. Queens can inhibit dopamine increases in workers by queen substance, whereas workers can manipulate dopamine levels by feeding amino acid-rich food containing tyrosine to queens, males, and other workers. Such manipulations of dopamine production can adjust the physiological states required for reproduction and maintain sex-specific behaviors in response to unpredictable environments.

## Supplementary Materials

The following supporting information can be downloaded at: https://www.mdpi.com/article/10.3390/insects13020128/s1, Table S1: Mean values of the monoamine levels and the ratios in the brains of workers and males under control and tyrosine-fed conditions.
