# Supplementary material for: Sex-Specific Regulatory Systems for Dopamine Production in the Honey Bee
Source: Insects. 2022 Jan 25;13(2):128. doi: 10.3390/insects13020128 (PMC8878259; doi:10.3390/insects13020128)
Supplement: Supplementary file 1 [file insects-13-00128-s001.zip › insects-1543637-supplementary.pdf]

Table S1. Mean values of monoamine levels and the ratios in the brains of workers and males under control and tyrosine-fed condition.

Monoamine	Worker (8 days)				Male (8 days)			
	Control	Tyrosine-fed (1mg/mL)	Tyrosine-fed / Control	Notes	Control	Tyrosine-fed (1mg/mL)	Tyrosine-fed / Control	Notes
DOPA	0.828 pmol / brain	1.571 pmol / brain	1.90	unpublished data	4.476 pmol / protein mg	13.576 pmol / protein mg	3.03	Ref. 3
Dopamine	21.084 pmol / brain	60.770 pmol / brain	2.88	Ref. 1	17.678 pmol / brain	39.063 pmol / brain	2.21	Ref. 2
	16.384 pmol / brain	34.548 pmol / brain	2.11	Ref. 2	110.446 pmol / protein mg	218.944 pmol / protein mg	1.98	Ref. 3
N-acetyldopamine	14.750 pmol / brain	35.247 pmol / brain	2.39	Ref. 1	13.754 pmol / brain	23.159 pmol / brain	1.68	Ref. 2
	8.094 pmol / brain	18.530 pmol / brain	2.29	Ref. 2				
Tyramine	2.187 pmol / brain	4.073 pmol / brain	1.86	Ref. 1	14.310 pmol / protein mg	12.651 pmol / protein mg	0.88	Ref. 3
Octopamine	2.226 pmol / brain	2.068 pmol / brain	0.93	Ref. 1	no data	no data	-	
N-acetyltyramine	9.971 pmol / brain	12.478 pmol / brain	1.25	Ref. 1	no data	no data	-	

Ref. 1: Matsuyama et al. (2015); Ref. 2: Sasaki (2016); Ref. 3: Watanabe and Sasaki (2021)
